# Inhibitors of Eicosanoid Biosynthesis Influencing the Transcripts Level of sHSP21.4 Gene Induced by Pathogen Infections, in *Antheraea pernyi*

**DOI:** 10.1371/journal.pone.0121296

**Published:** 2015-04-06

**Authors:** Congfen Zhang, Lishang Dai, Lei Wang, Cen Qian, Guoqing Wei, Jun Li, Baojian Zhu, Chaoliang Liu

**Affiliations:** 1 College of Life Science, Anhui Agricultural University, Anhui Hefei, P.R. China, 230036; 2 Department of Pharmacology, Wannan Medical College, Anhui Wuhu, P.R.China, 241002; Institute of Plant Physiology and Ecology, CHINA

## Abstract

Small heat shock proteins (sHSPs) can regulate protein folding and protect cells from stress. To investigate the role of sHSPs in the silk-producing insect *Antheraea pernyi* response to microorganisms, a sHsp gene termed as *Ap-sHSP21*.*4*, was identified. This gene encoded a 21.4 kDa protein which shares the conserved structure of insect sHsps and belongs to sHSP21.4 family. *Ap-sHSP21*.*4* was highly expressed in fat body and up-regulated in midgut and fat body of *A*. *pernyi* challenged with *Escherichia coli*, *Beauveria bassiana* and nuclear polyhedrosis virus (NPV), which was determined by quantitative real-time PCR. Meanwhile, knock down of *Ap-sHSP21*.*4* with dsRNA result in the decrease at the expression levels of several immune response-related genes (defensin, Dopa decarboxylase, Toll1, lysozyme and Kazal-type serine protease inhibitor). Additionally, the impact of eicosanoid biosynthesis on the expression of *Ap-sHSP21*.*4* response to NPV was determined using qPCR, inhibitors of eicosanoid biosynthesis significantly suppress *Ap-HSP21*.*4* expression upon NPV challenge. All together, *Ap-sHSP21*.*4* was involved in the immunity of *A*. *pernyi* against microorganism and possibly mediated by eicosanoids pathway. These results will shed light in the understanding of the pathogen-host interaction in *A*. *pernyi*.

## Introduction

Heat shock proteins (HSPs) are evolutionarily highly-conserved proteins synthesized in cells when they are exposed to stress, which are found in almost all organisms [1–4]. Heat shock proteins can be divided into five families, including *HSP100*, *HSP90*, *HSP70*, *HSP60*, and small heat shock proteins (12–42 kDa) according to approximate molecular weight and protein characters. sHSPs were discovered in the salivary glands of *Drosophila* after heat shock [5,6] and are closely associated with the duration of stresses [7–9] such as cellular communication, immune response, protein transport, apoptosis and cell cycle regulation [10–13].

Insects are more or less constantly challenged with a daunting array of pathogenic organisms, including viruses, bacteria, fungi, protozoans as well as various metazoan parasites and parasitoids. Eicosanoids mediate melanotic nodulation reactions to pathogens infection in larvae of *Pimpla turionellae* and *Bombyx mori* [14–17] found that eicosanoid biosynthesis inhibitors significantly repressed the induction of the cecropin and lysozyme genes elicited by peptidoglycan. It is reported that both cyclooxygenase and lipoxygenase products were involved in nodulation responses to bacterial infections [18,19] and phagocytosis [20]. On the other hand, HSP induction represents an important adaptive response to stress and is also associated with local increase in tissue temperature. In addition, Bundey et al [21] also reported that eicosanoids mediate behavioral fever responses to infection in the locust *Schistocerca gregaria*. Taking together, we propose that eicosainods pathway is involved the HSP gene transcription in insect challenged by pathogens.

The Chinese oak silk moth *Antheraea pernyi* (Lepidoptera: Saturniidae; *A*. *pernyi*) is an economically valuable silk-producing insect that is commercially cultivated mainly in China, India, and Korea [22]. The sHSP21.4 genes have been found in a variety of insects [23,18], however, the *HSP21*.*4* gene in the *A*. *pernyi* (*Ap-sHSP21*.*4*, GenBank accession number: KM881571) and its function remains unclear. In this study, we aimed to clone the genes of sHSP21.4 and investigate its role in the immune response against microorganisms.

## Materials and Methods

### Experimental insects and treatment

*A*. *pernyi* larvae were provided by the Sericultural Research Institute of Henan and were reared on oak leaves under indoor conditions. The larvae were reared on fresh oak leaves at 25 ± 1°C in 14 h light: 10 h dark (a long day length) with 70% humidity. Five 3^rd^ day fifth instar larvae were randomly sampled at each time point after exposure to infection. The total RNA extracted from hemocytes, fat bodies, and midguts of *A*. *pernyi* larvae after challenges with heat-killed bacteria (*Escherichia coli*, *E*. *coli* and *Beauveria bassiana*, *B*. *bassiana*), *A*. *pernyi* nuclear polyhedrosis virus (NPV) and control sample (1.0 ×10^6^ bacterial cells or 1.0 ×10^6^ fungal spores or 1.0×10^9^ virus particles were suspended in 10 μL of sterilized 0.85% NaCl, and then were separately injected into each larvae) [24]. Tissues were sampled for RNA extraction at 0 h, 3 h, 6 h, 12 h, 24 h, 48 h after infection and stored at -80°C and subjected to qPCR testing [25].

### RNA extraction, cDNA synthesis, PCR primers, and conditions

Total RNA was isolated from fat bodies with TRIzol reagent (Invitrogen, USA) and first-strand cDNA was obtained using TransScript Synthesis SuperMix (TransGen, Beijing, China). The *Ap-sHSP21*.*4* sequences from various animals were aligned by ClustalW (http://www.ebi.ac.uk/Tools/ClustalW). The degenerate oligonucleotide primers F2 and R2 (Table 1) were designed with Primer premier 5.0. PCR was performed using the amplification program with the following protocol: one cycle at 94°C for 5 min; followed by 35 cycles of 94°C for 30 s, 55°C for 35 s, and 72°C for 30 s; and a final elongation step of 72°C for 8 min. The PCR products were analyzed by 1% agarose gel electrophoresis, and sequenced at Invitrogen, Shanghai.

**Table 1:** Primers used in this study

Primer	sequences (5'–3')	Application
F1	CAACACAGCATAGCGATAGCAG	qPCR
R1	GATTTCTCCTCGTGTTTGGCAT	qPCR
F18S	CGATCCGCCGACGTTACTACA	qPCR
R18S	GTCCGGGCCTGGTGAGATTT	qPCR
F2	ATGGCTGACAGTGGTCTCAAG	amplification
R2	TCAGTGCTTCTGGATAGGAATG	amplification
F3	CGCGGATCC ATGGCTGACAGTGGTCTCAAG	expression
R3	ATAAGAATGCGGCCGCTCAGTGCTTCTGGATAGGAATG	expression
F4	TGCCAAACACGAGGAGA	RACE PCR
F 5	ACTCGCCATTACCGACAGG	RACE PCR
R4	TCTGCTATCGCTATGCTGT	RACE PCR
R5	TTTGCCGTCACCTTCATCC	RACE PCR

### Bioinformatics and phylogenetic analysis

NCBI bioinformatics tools (available at http://blast.ncbi.nlm.nih.gov/Blast.cgi) were used to detect the conserved domains in *Ap-sHSP21*.*4*. DNAman software was used to predict the secondary structure and search for open reading frame (ORF). Multiple sequence alignments were performed using ClustalX with its default parameters [26]. Phylogenetic analysis was performed using the neighbor-joining method with Molecular Evolutionary Genetics Analysis (MEGA version 6.0) software.

### Expression analysis using quantitative RT-PCR

The total RNA from hemocytes, fat body, midgut, epidermis, silk gland, and Malpighian tubules of 3^rd^ day fifth instar larvae were reverse transcribed into cDNAs. The housekeeping gene 18S rRNA (GenBank: DQ347469) was used as an internal control. RT- PCR was performed in a StepOne Plus Real-Time PCR System using the SYBR Premix Ex Taq kit (TaKaRa) with the specific primers in Table 1. A 20 μL reaction mixture contained 10 μL of 2× SYBR Premix Ex Taq buffer, 1 μL each of forward and reverse primers, 1 μL cDNA, and 7 μL Rnase-free H_2_O. The PCR procedure was as follows: 95°C for 10 s followed by 40 cycles each at 95°C for 15 s, 62°C for 15 s, and 72°C for 30 s. At the end of the reaction, a melting curve was produced by monitoring the fluorescence continuously while slowly heating the sample from 60°C to 95°C. Each independent experiment was conducted in triplicate and the data were analyzed using ANOVA method. It was considered statistically significant when *P* value less than 0.05 and the significance was indicated by an asterisk [27].

### RNA interference of *Ap-sHSP21*.*4* gene

The siRNAs (Table 1) were designed by the siRNA Selection Program (http://sirna.wi.mit.edu/home.php) and chemically synthesized by Shanghai GenePharma Co., Ltd. (Shanghai, China). The BLAST homology search (http://www.ncbi.nlm.nih.gov/BLAST) was performed to avoid off-target effects on other genes or sequences. The siRNAs were purified by high-performance liquid chromatography and were dissolved in diethylpyrocarbonate-treated water (Milli-Q-grade). The final concentration of siRNA was 1 μg/μL H_2_O. The 10 μL of siRNA was injected into each larva using microliter syringes (Gaoge Co., Shanghai, China). To avoid leakage of siRNA from the insect body, needles were kept still at the injection point for 30 s. One set of siRNAs with random sequences was used as a negative control and injected alongside the experimental injection. Twenty-one or fourty-five hours after RNAi treatment, the larvae were injected with NPV (1.0×109 virus particles/larvae) followed by recovery at 25°C for 3 h. The fat bodies of the larvae were collected at 24 and 48 h after siRNA injection, frozen in liquid nitrogen and stored at -80°C. All experiments were conducted with two independent experiments in triplicate.

### Effect of inhibitors of arachidonic acid on the expression of *Ap-sHSP21*.*4* against NPV

All inhibitors and arachidonic acid were purchased from Sigma Chemicals. Test larvae were anesthetized with CO_2_ and first injected with inhibitor (phospholipase A2 inhibitor dexamethasone [DEX], lipoxygenase inhibitor nordihydroguaiaretic acid [NDGA], and cyclooxygenase inhibitor indomethacin [INDO]), arachidonic acid (AA) in 10 μL of DMSO, or DMSO alone (control). After incubating the mixtures for 30 min at room temperature, the larvae received a second injection of NPV (1.0×10^9^ virus particles /larvae) or PBS alone, and were further incubated at 27°C. The fat body and midgut were collected at 3 h or 24 h respectively after the second injection, rinsed in ice-cold PBS solution, and frozen on dry ice [17].

## Results

### Cloning and sequence analysis of *Ap-sHSP21*.*4* cDNA

The full-length cDNA of *Ap-sHSP21*.*4* was 1,391 bp in length and encoded 187 amino acids with a calculated molecular mass of 21.4 kDa (S1A Fig).

This predicted protein shared 97.72, 97.26, and 97.26% identity with sHSP21.4 proteins from *Bombyx mori*, *Helicoverpa armigera* and *Chilo suppressalis*. It represents a conserved C-terminal domain of -crystallin-type sHSPs and belongs to the lepidopteran HSP21.4 family (S1B Fig). However, most insect sHSPs are species-specific. This strengthens the hypothesis that sHSP21.4 might have followed another evolutionary course [28].

### Expression of *Ap-sHSP21*.*4* in tissues and under microorganism challenges

**Fig 1:**
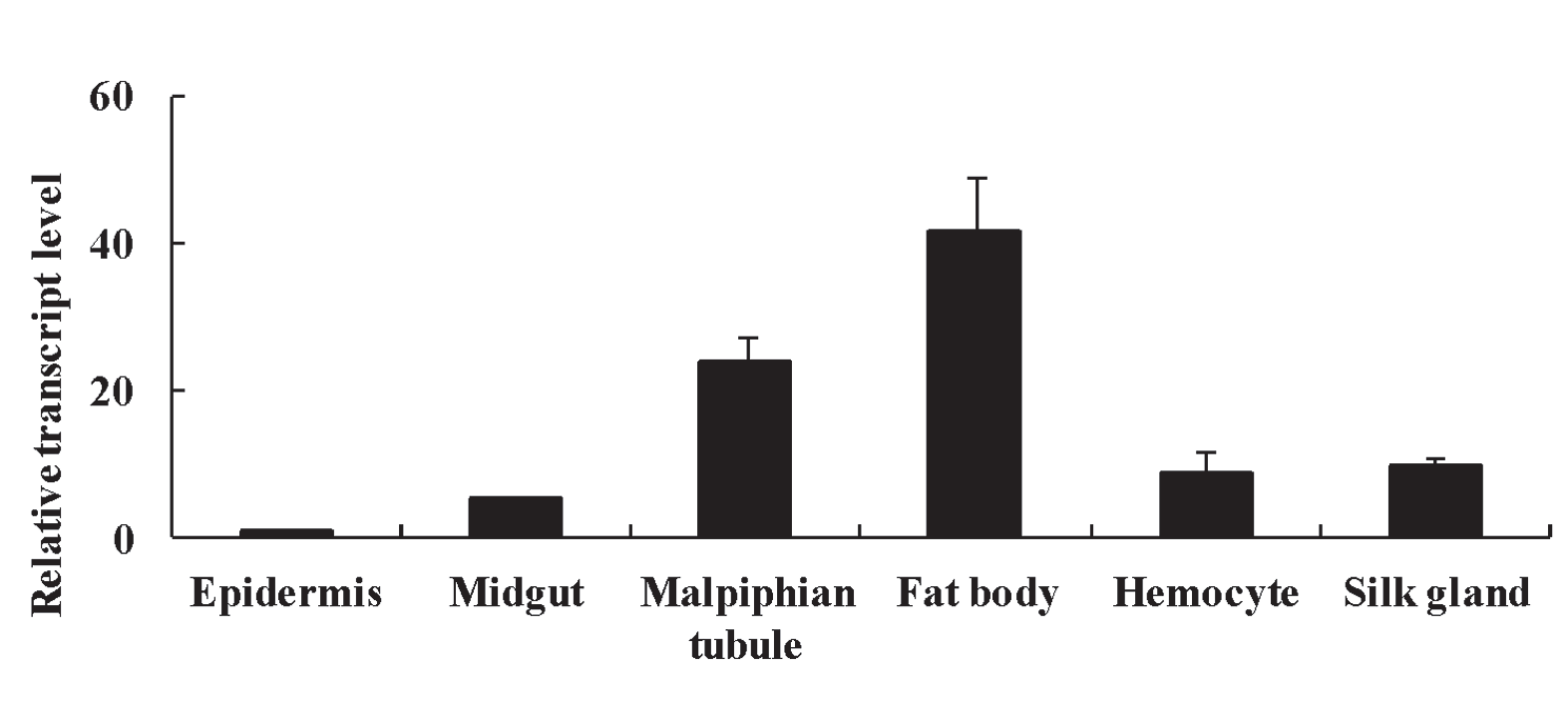
The expression of *Ap-sHSP21*.*4* in different tissues and cells.

The sHSP gene was commonly expressed in all examined tissues (Fig 1).

**Fig 2:**
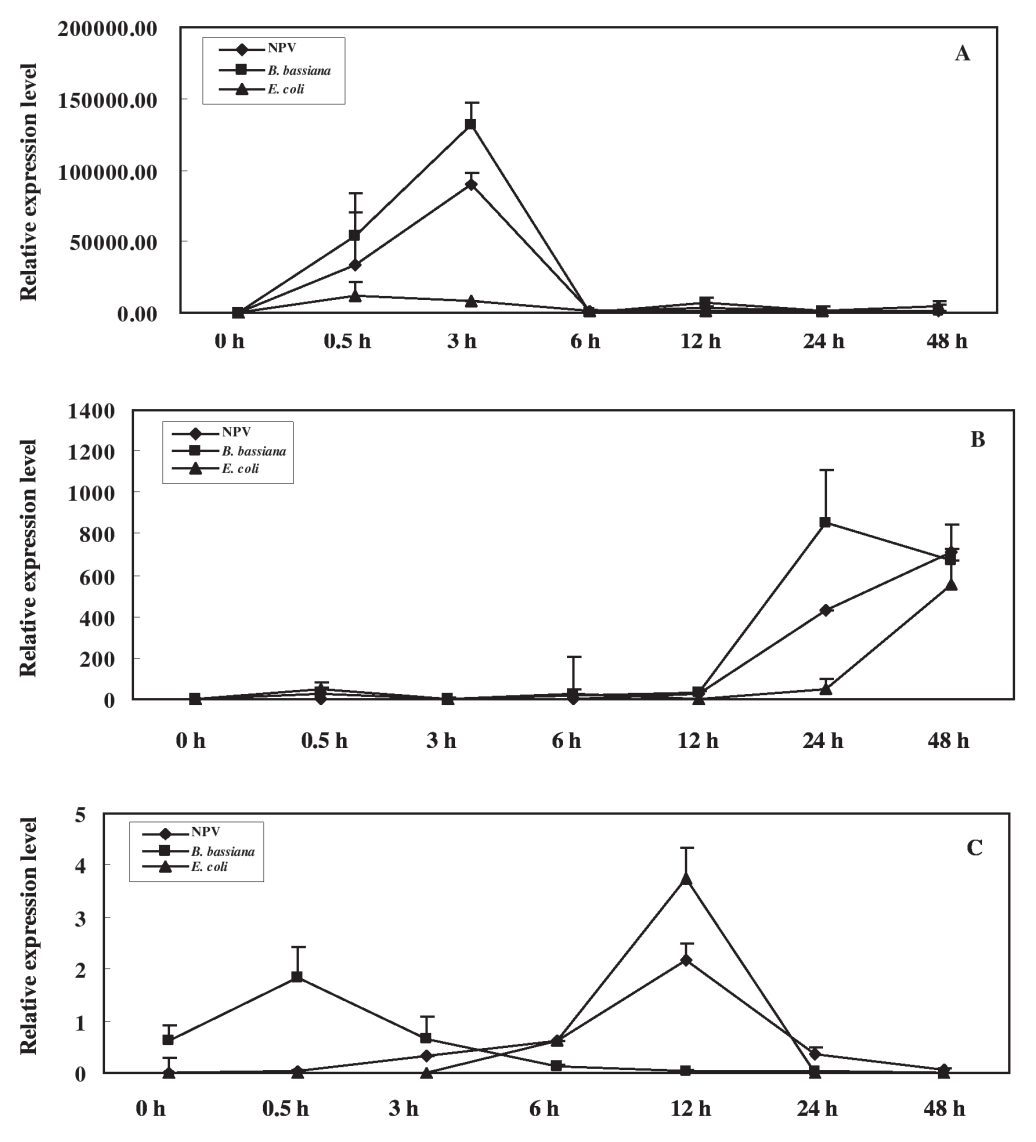
Expression patterns of the *Ap-sHSP21*.*4* under microorganism challenges in different tissues (five larvae for each group). **(A):** the expression of *Ap-sHSP21*.*4* in the fat body, **(B):** the expression of *Ap-sHSP21*.*4* in the midgut. **(C):** the expression of *Ap-sHSP21*.*4* in the hemocytes.

The expression level of *Ap-sHSP21*.*4* transcript was highest in the fat body and lowest was epidermis. To investigate whether *Ap-sHSP21*.*4* is involved in the immune response, three types of microorganisms were individually injected into the bodies of fifth-instar larvae. The *Ap-sHSP21*.*4* transcripts quickly accumulated in the fat body and reached a maximum at 3 h, and then gradually decreased. The *Ap-sHSP21*.*4* gene was found to be more susceptible to NPV and *B*. *bassiana* compared with *E*. *coli* in fat bodies (Fig 2A).

In the midgut, the expression of *Ap-sHSP21*.*4* was increased at 24 h after injected with NPV, *B*. *bassiana* or *E*. *coli* (Fig 2B). In the hemocytes, the expression of the *Ap-sHSP21*.*4* showed a similar trend under NPV and *E*. *coli* challenge, and the highest expression level was detected at 12 h after the challenge, whereas the gene expression showed an almost immediate response to *B*. *bassiana* challenge (Fig 2C). These results suggest that *Ap-sHSP21*.*4* has different expression patterns in various tissues upon different microorganisms.

### RNAi of *Ap-sHSP21*.*4* influences the expression of immune response-related genes

**Fig 3:**
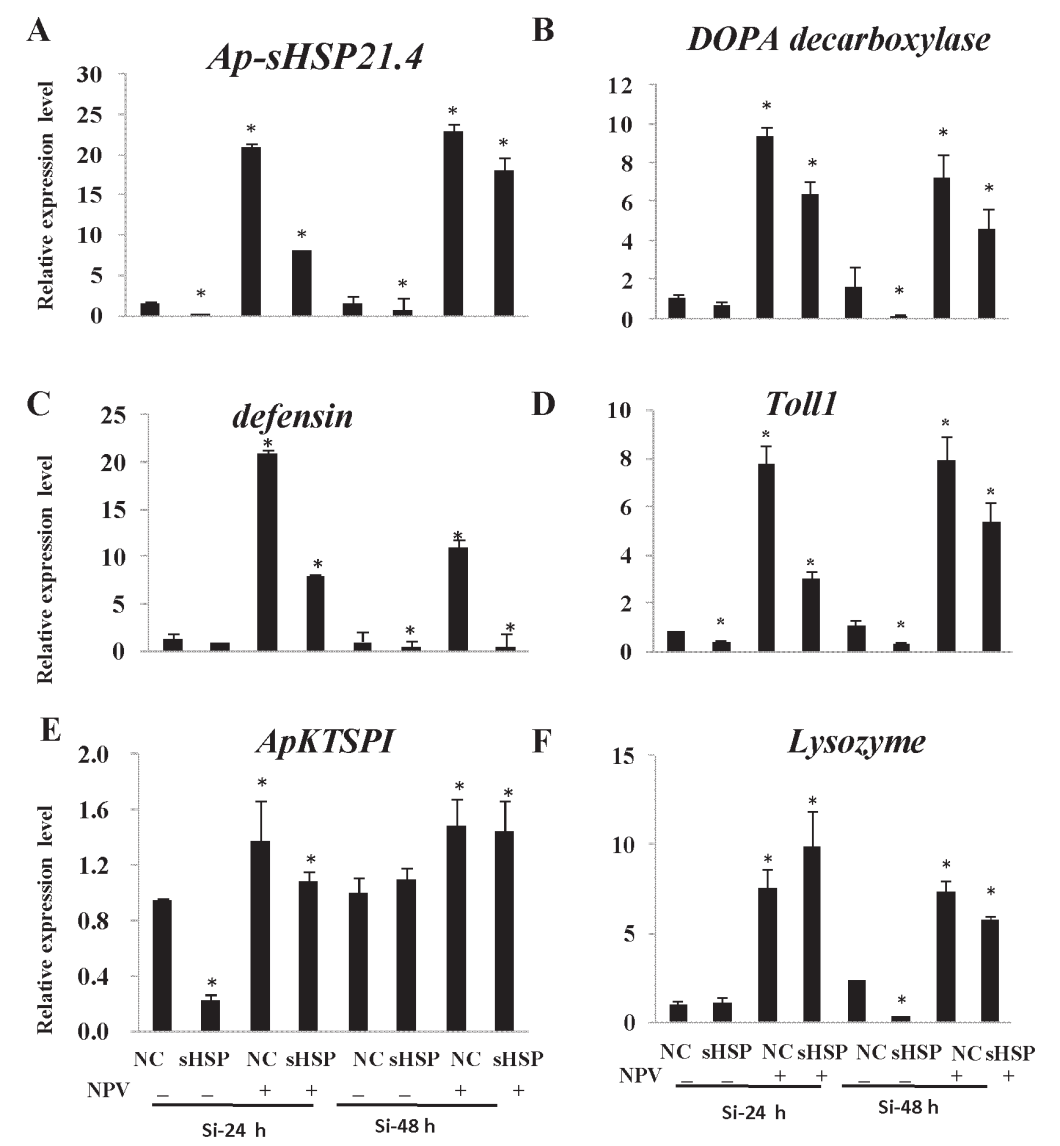
Relative mRNA expression levels of *Ap-sHSP21*.*4* and immune response-related genes in response to the NPV stress by injection of siRNA at 24 and 48 h. Expression levels were assessed using 18S rRNA gene for normalization. One set of negative control siRNAs (si-NC) was injected alongside the experimental injections. The data were analyzed by ANOVA and presented as mean ± SE of independent experiments done in triplicate and the asterisk represents the significant differences.

The expression level of *Ap-sHSP21*.*4* in the fat bodies of *A*. *pernyi* larvae injected with siRNAs is significantly decreased compared with controls. When normalized to the housekeeping gene 18sRNA, the relative expression levels of *Ap-sHSP21*.*4* dropped to 20% or 64% of the control values at 24 h and 48 h after siRNA injection (Fig 3A). This indicated that the expression of *Ap-sHSP21*.*4* gene in *A*. *pernyi* was inhibited successfully when RNAi was administered by injection. The expression levels of *Ap-sHSP21*.*4* were up-regulated significantly after injected with NPV at 24 and 48 h.

To further validate the transcripts level of immune response-related genes after si-*Ap-sHSP21*.*4* injection, qRT-PCR was used to detect the transcription levels of the selected immune response-related genes in fat bodies. The transcription levels of most immune response-related genes (defensin, Dopa decarboxylase, Toll1, lysozyme and Kazal-type serine protease inhibitor) increased at least two fold compared to the control after NPV infection (Fig 3A). The transcription level of defensin significantly increased about 20-fold and Dopa decarboxylase 9 had the highest transcription level after NPV injection, while the expression levels down-regulated to 0.55 and 0.15-fold by treatment with si-*Ap-sHSP21*.*4* (Fig 3B and 3C). The transcription levels of Toll1 were strongly induced almost 8-fold, then reduced to 0.28-fold by injection of *Ap-sHSP21*.*4* dsRNA (Fig 3D). The transcription level of ApKTSPI reached the peak with 1.4-fold under NPV injection and decreased to 0.22-fold after RNA interference (Fig 3E), this result is similar to that for lysozyme (Fig 3F). All these data suggested that *Ap-sHSP21*.*4* was probably involved in immune regulation.

### Eicosanoid mediated the induction of *Ap-sHSP21*.*4 *by NPV

**Fig 4:**
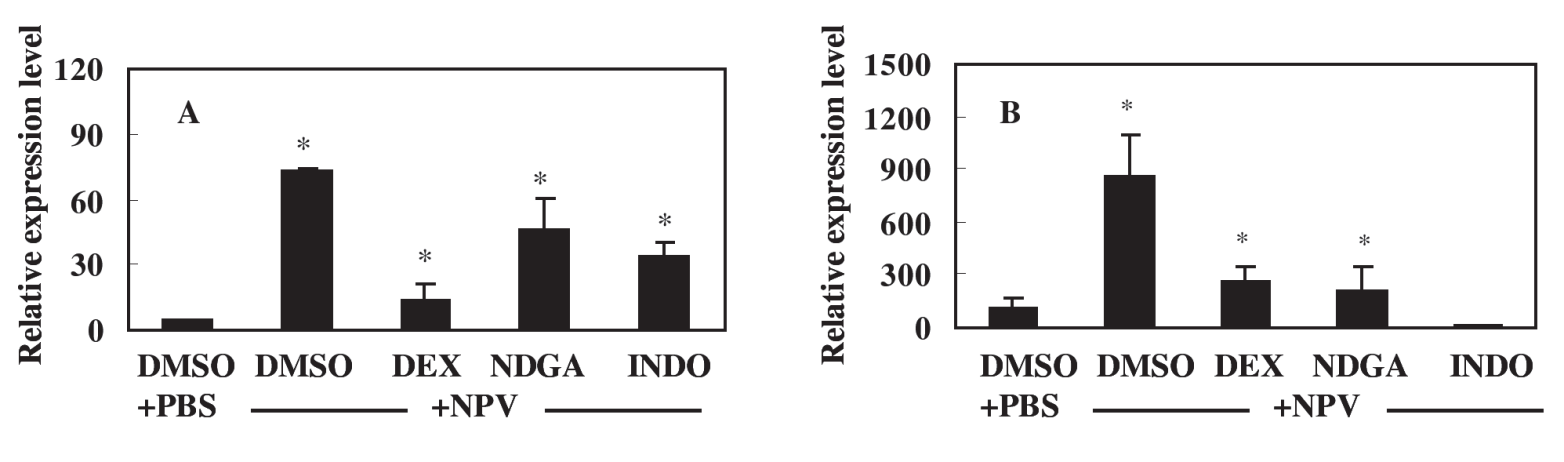
Effect of eicosanoid biosynthesis inhibitors on the expression of *Ap-sHSP21*.*4* in response to NPV. The larvae were at first injected with different concentrations (in 10 μL DMSO) of either dexamethasone (DEX), nordihydroguaiaretic acid (NDGA), or indomethacin (INDO). Control larvae were injected with 10 μL of DMSO alone. After keeping the larvae at room temperature for 30 min, the larvae then received a second injection of either insect PBS or 10 μL of NPV (10^9^ /larva). Total RNA was isolated from the fat body (A) and midgut (B) 3 h or 24 h respectively after the second injection, and the *Ap-sHSP21*.*4* gene expression was analyzed by quantitative real-time PCR. The mRNA levels are shown as a percentage of the levels in the DMSO control larvae. Bars represent the means ± SD (n = 5).

To study the effect of eicosanoid biosynthesis inhibitors on the induction of *Ap-sHSP21*.*4* by NPV, silkworm larvae were firstly injected with drugs, then challenged with NPV. Three pharmaceutical inhibitors of eicosanoid biosynthesis dramatically suppress *Ap-HSP21*.*4* expression upon NPV challenge (Fig 4).

For example, the relative transcript level of *Ap-HSP21*.*4* declined dramatically in a statistically manner from approximately 76-fold (treated with NPV) to 14-fold (treated with o.1 M DEX +NPV). The similar declines in relative transcript level of *Ap-HSP21*.*4* were found in groups treated with INDO and NDGA. Furthermore, *Ap-sHSP21*.*4* was significantly induced in the fat body and midgut by NPV injection. All the inhibitors injected prior to the treatment with NPV greatly suppressed the NPV-induced expression of the *Ap-sHSP21*.*4*.

### Induction of *Ap-sHSP21*.*4* by arachidonic acid

**Fig 5:**
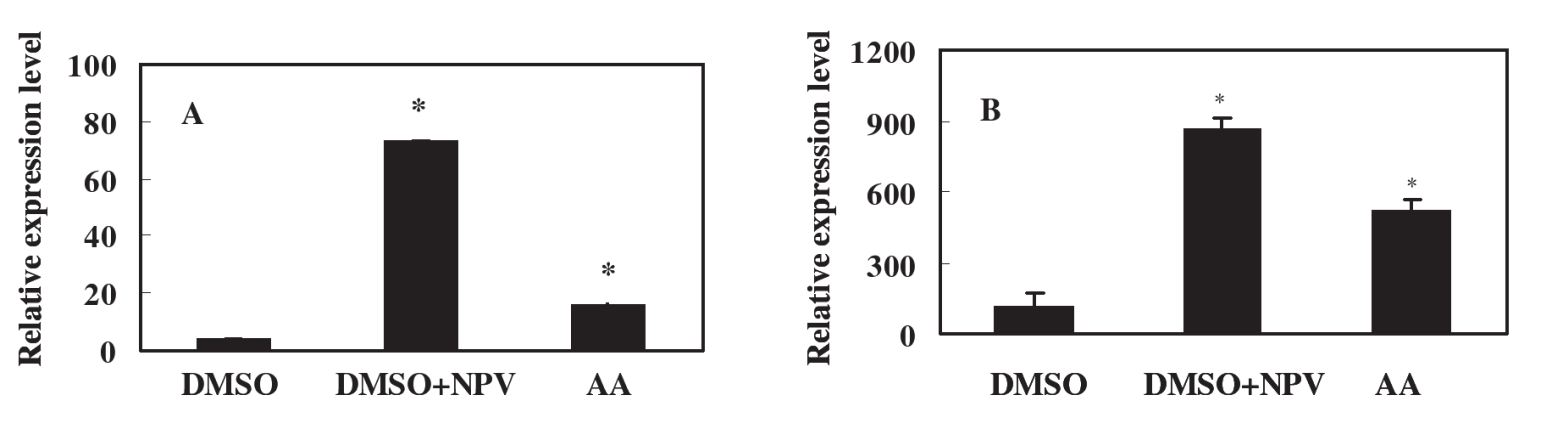
Induction of *Ap-sHSP21*.*4* by arachidonic acid in the fat body (A) and midgut (B). The larvae were injected with either 10 μL of DMSO (control), 10 μL of NPV (109 /larva) or arachidonic acid in 10 μL of DMSO (AA). The fat body (A) and midgut (B) 3 h or 24 h respectively after the second injection, The mRNA levels are shown as a percentage of the levels in the DMSO-treated control larvae. Bars represent the means ± SD (n = 5).

The previous results strongly suggested that arachidonic acid metabolites mediated the induction of immune gene in the fat body, hemocyte and midgut. To test the direct effect of arachidonic acid metabolites on the induction, the larvae were treated with arachidonic acid, and the *Ap-sHSP21*.*4* mRNA levels in the fat body and midgut were examined. As shown in Fig 5, the arachidonic acid could induce the gene expression, although the levels were somewhat lower than that induced by NPV. The increase in *Ap-sHSP21*.*4* gene expression by arachidonic acid was significantly up-regulation (*P*>0.05) compared to that in the control (treated with DMSO).

## Discussion

sHSPs are a highly conserved, ubiquitously expressed family of proteins whose synthesis is induced in response to heat shock or adverse environments. Here, we identified a sHSP21.4 gene from *A*. *pernyi*. Multiple sequence alignment revealed that this sHSP from *A*. *pernyi* was highly homologous with those from other insects, suggesting that they may have a similar function to the sHSPs in the other species. The phylogenetic analysis of these superfamilies supports the traditional morphology-based classification [29].

Despite the main function of HSPs is to protect proteins from stress conditions, accumulating studies have shown that they are also involved the anti-biotic stress response. The expression level of *Ap-sHSP21*.*4* was more abundant in the fat body than other tissues, which may indicate that the fat body was more sensitive to biotic stresses. In this experiment, the expression of *Ap-sHSP21*.*4* was up-regulated by three types of microorganisms (bacteria, virus and fungi) challenges in the fat body and midgut. However, the expression patterns of *Ap-sHSP21*.*4* varied in different tissues challenged with various microorganisms. For example, challenged with NPV and *B*. *bassiana* induced acute *Ap-sHSP21*.*4* gene expression accumulation at 3 h pt with nearly 2 millions-fold in the fat body compared with the control (Fig 5A). But the response was much slower in the midgut, which appeared after 24 hours (Fig 5B). It is considered that these differential effects of sHSP upon different pathogens injection may be caused by their respective signaling pathway [30]. To further verify the role of *Ap-sHSP21*.*4* in immune response, several immune response-related genes (defensin, decarboxylase, Toll, lysozyme and serine protease inhibitor) were selected for detection after si-*Ap-sHSP21*.*4* injection. The transcription levels of most immunity response genes increased compared with the control after NPV infection (Fig 3A), while were significantly suppressed after RNAi *Ap-sHSP21*.*4*. The pattern of regulations is similar to Beetle, in which septic injury induces expression of genes involved in stress adaptation (e.g. heat-shock proteins) or potential antimicrobial effectors (e.g. ferritin, c-type lysozyme, serine proteinase inhibitors, and defensins), suggesting that there may be crosstalk between the immune and stress responses [11]. These results indicate the *Ap-sHSP21*.*4* plays a role in immune response in *A*. *pernyi* against microorganism.

Aspirin and other non-steroidal anti-inflammatory drugs (NSAIDs) that act by inhibiting COX (the core enzyme in PG biosynthesis) have been studied in many areas of human and veterinary pathophysiology. Stanley-Samuelson and his colleagues have extensively studied the effects of pharmacological inhibitors of eicosanoid metabolism in insects [31–33]. The tobacco hornworm *Manduca sexta* injected with pharmaceutical inhibitors of eicosanoid biosynthesis (NSAIDs) could impair their ability to clear injected bacteria from hemolymph circulation [34]. Carton et al. reported that the PLA2-inhibiting glucocorticoid dexamethasone inhibited the encapsulation of parasitoid eggs in a resistant line of *Drosophila melanogaster* [35]. In the fat body of *B*. *mori*, specific inhibitors of phospholipase A2, cyclooxygenase, and lipoxygenase significantly inhibited the induction of the immune genes both in vivo and in cultured fat bodies. Arachidonic acid has been also found to induce the expression of the cecropin and lysozyme genes [17]. Our data clearly show that eicosanoid biosynthesis inhibitors suppress the expression of the *Ap-sHSP21*.*4* elicited by NPV, while arachidonic acid induces the expression of *Ap-sHSP21*.*4* in the fat body and midgut. All these suggest eicosanoid mediates the response of *Ap-sHSP21*.*4* against immune challenges.

## Supporting Information

S1 Fig Amino acid sequence comparison (A) and phylogenetic analysis of sHSP amino acid sequences from different insect species (B). A: The sHSPs proteins are from *H*. *armigera* (AGC39039.1), *H*. *erato* (ABS57447.1), *C*. *suppressalis* (AGC23338.1), *B*. *mori* (NM_001043520.1), *E*. *pela* (AGE92593.1), *L*. *migratoria* (ABC84493.1), *A*. *darling* (ETN64726.1), *R*. *pedestris* (BAN20225.1), *M*. *domestica* (NP_001273840.1). B: phylogenetic tree was constructed using the neighbor-joining algorithm method with a bootstrap test of 1000 repetitions.
(TIF)
