# Supplementary figures and images for: Inhibitors of Eicosanoid Biosynthesis Influencing the Transcripts Level of sHSP21.4 Gene Induced by Pathogen Infections, in Antheraea pernyi
Source: PLoS One. 2015 Apr 6;10(4):e0121296. doi: 10.1371/journal.pone.0121296 (PMC4386827; doi:10.1371/journal.pone.0121296)

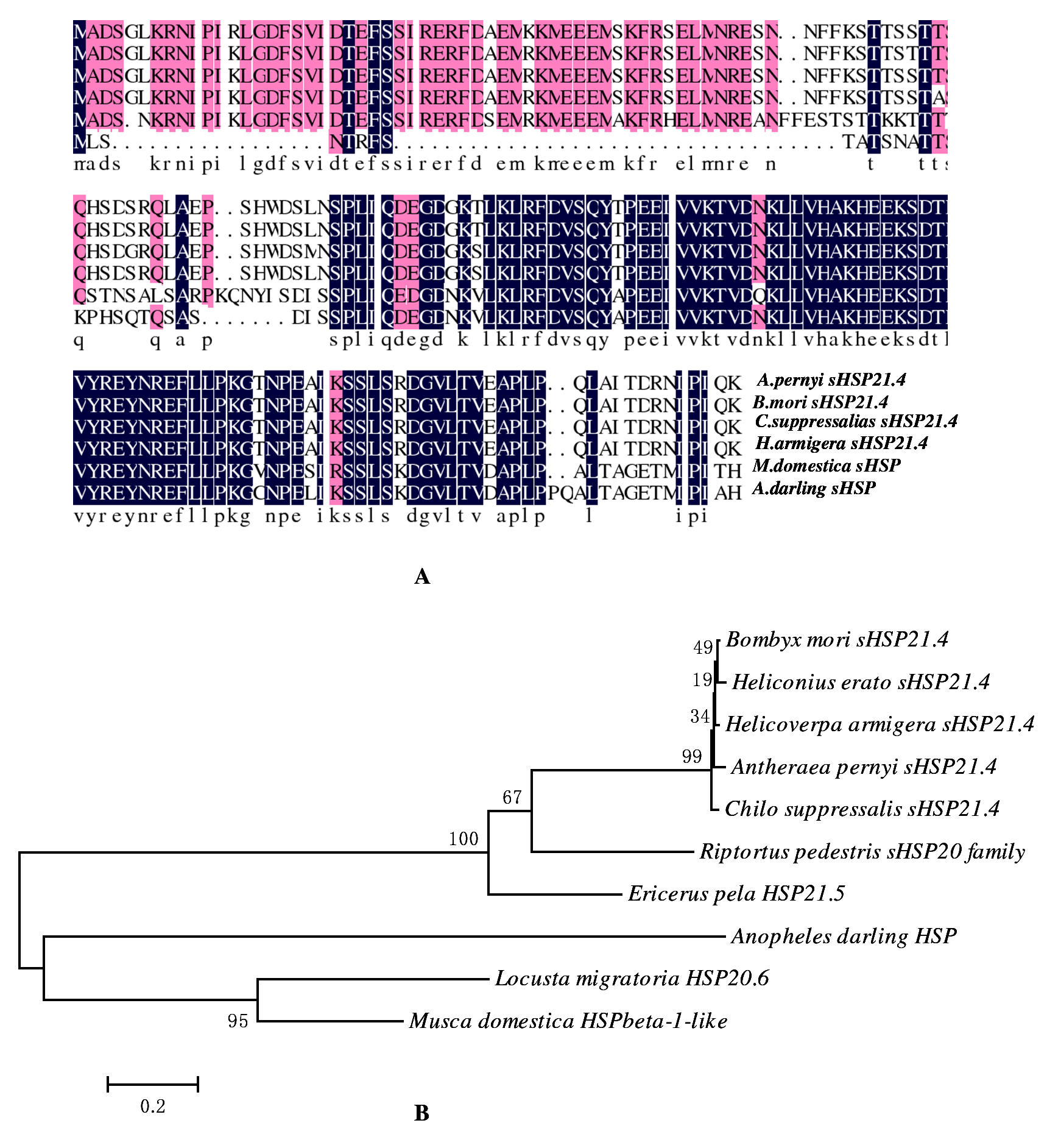

Supplement: S1 Fig — A: The sHSPs proteins are from H. armigera (AGC39039.1), H. erato (ABS57447.1), C. suppressalis (AGC23338.1), B. mori (NM_001043520.1), E. pela (AGE92593.1), L. migratoria (ABC84493.1), A. darling (ETN64726.1), R. pedestris (BAN20225.1), M. domestica (NP_001273840.1). B: phylogenetic tree was constructed using the neighbor-joining algorithm method with a bootstrap test of 1000 repetitions. (TIF) [file pone.0121296.s001.tif]
